# Novel Levofloxacin-Resistant Multidrug-Resistant *Streptococcus pneumoniae* Serotype 11A Isolates, South Korea

**DOI:** 10.3201/eid2211.151450

**Published:** 2016-11

**Authors:** Miey Park, Hyun Soo Kim, Han-Sung Kim, Ji Young Park, Wonkeun Song, Hyoun Chan Cho, Jae-Seok Kim

**Affiliations:** Hallym University College of Medicine, Seoul, South Korea

**Keywords:** Streptococcus pneumoniae, multidrug-resistant, MDR, levofloxacin-resistant, serotype 11A, bacteria, South Korea, antimicrobial resistance, streptococci, respiratory infections

## Abstract

Of 608 *Streptococcus pneumoniae* clinical strains isolated at a hospital in South Korea during 2009–2014, sixteen (2.6%) were identified as levofloxacin resistant. The predominant serotype was 11A (9 isolates). Two novel sequence types of multidrug-resistant *S. pneumoniae* with serotype 11A were identified, indicating continuous diversification of resistant strains.

*Streptococcus pneumoniae* is a common respiratory pathogen that is the leading cause of community-acquired pneumonia (*1*). Although β-lactam antibiotics have long been used for the treatment of respiratory diseases, the increasing prevalence of antibiotic-resistant *S. pneumoniae* strains has hampered treatment in recent decades (*2*,*3*). Resistance to fluoroquinolones has emerged in *S. pneumoniae* and is caused by mutations within short DNA sequences of *gyrA* and *parC* genes that encode the type II topoisomerase subunits known as quinolone-resistance determining regions (QRDRs) (*1*). Previous studies have shown that most of the *S. pneumoniae* strains with reduced susceptibility to the fluoroquinolone levofloxacin exhibit a multidrug-resistant (MDR) phenotype (*2*,*4*). Levofloxacin resistance was closely associated with epidemic MDR clones (*3*). Although fluoroquinolone resistance rates remain low in *S. pneumoniae* in most countries, some extensively drug-resistant (XDR) *S. pneumoniae* isolates have emerged; this resistance is defined as nonsusceptibility to >1 agent in all but <2 antimicrobial categories (*2*,*4*). We examined *S. pnemoniae* isolates from patients in South Korea to determine antimicrobial resistance. We found novel sequence types (STs) of MDR serotype 11A *S. pneumoniae* that exhibit resistance to second-line antibiotics such as levofloxacin, ceftriaxone, and meropenem.

## The Study

During January 2009–December 2014, we isolated 608 *S. pneumoniae* clinical strains at a 698-bed, university-affiliated hospital in South Korea. We determined MICs by using the broth microdilution method according to Clinical and Laboratory Standards Institute guidelines (*5*). We performed antimicrobial resistance tests for levofloxacin, ofloxacin, ciprofloxacin, penicillin, amoxicillin, ceftriaxone, meropenem, erythromycin, clindamycin, vancomycin, linezolid, tetracycline, and tigecycline. We used *S. pneumoniae* ATCC 49619 as a control strain. We defined MDR as resistance or intermediate resistance to >3 antimicrobial agents.

We determined serotypes by using the multiplex PCR assay recommended by the Centers for Disease Control and Prevention (http://www.cdc.gov/ncidod/biotech/strep/pcr.htm). Reactions also included an internal positive control targeting all known pneumococcal *cpsA* regions (*6*). We sequenced QRDRs of the *gyrA*, *gyrB*, *parC*, and *parE* genes in each isolate (*7*). We performed multilocus sequence typing to investigate the genetic backgrounds of fluoroquinolone-resistant pneumococci (*8*) and assigned allele numbers and STs by using the PubMLST database (http://pubmlst.org/spneumoniae).

Of the 608 clinical *S. pneumoniae* isolates, 16 (2.6%) were levofloxacin resistant (MIC >8 μg/mL). We collected 1 resistant isolate in 2009, 3 in 2012, 5 in 2013, and 7 in 2014. Thirteen isolates were from sputum, and 3 isolates were from bronchial lavage. The mean age of patients was 71 years; 14 were male, and 2 were female.

Serotype 11A (n = 9) was most common among the levofloxacin-resistant isolates, followed by serotypes 13 (n = 2), 19F (n = 2), 23F (n = 2), and 6B (n = 1) (Table 1). The most common STs were ST9875 (n = 5), ST8279 (n = 3), and ST9876 (n = 3), which together accounted for 11 of the 16 levofloxacin-resistant isolates. Nine isolates of ST9875, ST9876, and ST10300 were novel STs and had not been identified before this study. 

**Table 1:** Select characteristics of 16 levofloxacin-resistant *Streptococcus pneumoniae* clinical isolates identified from patients at a hospital in South Korea, 2009–2014*†

Strain	Age, y/sex of patient	Specimen type	Respiratory disorders	Underlying disorders	Serotype	Sequence type
HM-646	36/M	Sputum	Pneumonia	CVA	11A	9875‡
HM-669	70/M	Sputum	Pneumonia	CVA	11A	9875‡
HM-683	77/M	Sputum	Pneumonia	COPD	6B	3173
HM-688	81/M	Sputum	Pneumonia	Cardiac infarction	23F	9876‡
HM-730	77/M	Sputum	Dyspnea with fever	Cervical pain	13	189
HM-762	76/F	Sputum	Pneumonia	Lung cancer	13	8279
HM-781	70/M	Sputum	Pneumonia	CVA	23F	6721
HM-787	35/M	Sputum	Pneumonia	CVA	11A	9875‡
HM-809	58/M	Sputum	Pneumonia	CVA	11A	9875‡
HM-854	77/M	BL	Pneumonia	Lung cancer	11A	99
HM-878	67/M	BL	Pneumonia	ALS	11A	8279
HM-953	82/M	Sputum	Pneumonia	COPD	11A	9875‡
HM-970	68/M	Sputum	Dyspnea with fever	Bronchiectasis	19F	9876‡
HM-1017	85/M	BL	Dyspnea	Lung cancer	11A	8279
HM-1050	62/M	Sputum	Postop atelectasis	CVA	19F	9876‡
HM-1055	89/F	Sputum	Pneumonia	CVA	11A	10300‡

*ALS, amyotrophic lateral sclerosis; BL, bronchial lavage; COPD, chronic obstructive pulmonary disorder; CVA, cerebrovascular accident. †Among the 16 isolates, 1 (HM-646) was collected in 2009; 3 (HM-669, HM-683, and HM-688) in 2012; 5 (HM-730, HM-762, HM-781, HM-787, and HM-809) in 2013; and 7 (HM-854, HM-878, HM-953, HM-970, HM-1017, HM-1050, and HM-1055) in 2014. ‡Novel sequence type found in our study.

All 16 levofloxacin-resistant isolates contained at least 2 amino acid alterations in the QRDRs of the *gyrA, parC*, and *parE* genes. Four QRDR mutations occurred with high frequency: Ser81Phe in *gyrA* was present in all 16 isolates; Ser79Phe and Lys137Asn in *parC* were present in 14 and 11 isolates, respectively; and Ile460Val in *parE* was found in 15 isolates. However, Lys137Asn in *parC* and Asp435Val and Ile460Val in *parE* are mutations not involved in resistance, according to previous reports (*9*,*10*). Isolate HM-854, which was penicillin susceptible, had Ser81Phe in *gyrA* and Asp79Asn in *parC* mutations. All isolates had >1 mutation in *parC*. The 2 isolates without the Ser79Phe mutation in *parC* instead carried Asp83Gly or Asp83Asn. The 4 isolates without the Lys137Asn mutation in *parC* instead carried the Asn91Asp mutation. Isolate HM-1017 (serotype 11A, ST-8279) had 7 QRDR mutations and exhibited the highest resistance against all antimicrobial agents, including levofloxacin (MIC 64 μg/mL). ST-8279 was associated with 2 different serotypes, 11A (n = 2) and 13 (n = 1). The 3 isolates of novel ST-9876 had the same QRDR amino acid changes but had different serotypes, 19F (n = 2) and 23F (n = 1).

The 16 levofloxacin-resistant isolates were also resistant to ofloxacin (MIC >8 μg/mL) and ciprofloxacin (MIC >8 μg/mL) (Table 2). All isolates except 3 had MICs >16 μg/mL against amoxicillin and ceftriaxone. Fourteen isolates were meropenem-resistant (MIC >1 μg/mL); all these isolates were susceptible to vancomycin and linezolid. Only 3 STs (ST-99, ST-189, and ST-3173) exhibited the lowest levofloxacin MIC (8 μg/mL); all these isolates were susceptible to amoxicillin (MIC <2 μg/mL).

**Table 2:** Antimicrobial susceptibilities of 16 levofloxacin-resistant *Streptococcus pneumoniae* clinical isolates identified from patients at a hospital in South Korea, 2009–2014*

Strain	MIC, μg/mL (resistance)												
	LEV	OFL	CIP†	PEN	AMX	CRO	MER	ERY	CLI	VAN	LZD	TET	TIG†
HM-646	16 (R)	32 (R)	32	>16 (R)	>16 (R)	>16 (R)	16 (R)	>16 (R)	>16 (R)	0.5 (S)	1 (S)	>16 (R)	0.03
HM-669	16 (R)	32 (R)	32	>16 (R)	>16 (R)	>16 (R)	8 (R)	>16 (R)	>16 (R)	0.5 (S)	1 (S)	>16 (R)	0.03
HM-683	8 (R)	16 (R)	16	4 (I)	2 (S)	2 (I)	1 (R)	>16 (R)	>16 (R)	0.5 (S)	1 (S)	>16 (R)	0.03
HM-688	16 (R)	32 (R)	32	>16 (R)	>16 (R)	>16 (R)	8 (R)	>16 (R)	>16 (R)	0.5 (S)	1 (S)	>16 (R)	0.03
HM-730	8 (R)	16 (R)	8	4 (I)	2 (S)	2 (I)	0.5 (I)	>16 (R)	>16 (R)	0.5 (S)	0.5 (S)	>16 (R)	0.03
HM-762	32 (R)	64 (R)	32	>16 (R)	>16 (R)	>16 (R)	16 (R)	>16 (R)	>16 (R)	0.5 (S)	0.5 (S)	>16 (R)	0.015
HM-781	16 (R)	32 (R)	16	16 (R)	16 (R)	>16 (R)	8 (R)	>16 (R)	>16 (R)	0.5 (S)	0.5 (S)	16 (R)	0.03
HM-787	16 (R)	32 (R)	64	16 (R)	16 (R)	>16 (R)	8 (R)	>16 (R)	>16 (R)	0.5 (S)	1 (S)	>16 (R)	0.03
HM-809	16 (R)	32 (R)	64	16 (R)	>16 (R)	>16 (R)	4 (R)	>16 (R)	>16 (R)	0.5 (S)	1 (S)	>16 (R)	0.03
HM-854	8 (R)	16 (R)	16	0.06 (S)	0.06 (S)	0.5 (S)	<0.015 (S)	8 (R)	0.06 (S)	0.5 (S)	1 (S)	>16 (R)	0.03
HM-878	16 (R)	32 (R)	32	16 (R)	>16 (R)	>16 (R)	8 (R)	>16 (R)	>16 (R)	0.5 (S)	1 (S)	4 (R)	0.03
HM-953	16 (R)	32 (R)	64	>16 (R)	>16 (R)	>16 (R)	16 (R)	>16 (R)	>16 (R)	0.5 (S)	1 (S)	>16 (R)	0.03
HM-970	32 (R)	64 (R)	32	>16 (R)	>16 (R)	>16 (R)	16 (R)	>16 (R)	>16 (R)	0.5 (S)	1 (S)	>16 (R)	0.03
HM-1017	64 (R)	128 (R)	64	>16 (R)	>16 (R)	>16 (R)	16 (R)	>16 (R)	>16 (R)	0.5 (S)	1 (S)	>16 (R)	0.03
HM-1050	32 (R)	64 (R)	64	>16 (R)	>16 (R)	>16 (R)	16 (R)	>16 (R)	>16 (R)	0.5 (S)	1 (S)	0.5 (S)	0.03
HM-1055	16 (R)	32 (R)	128	>16 (R)	>16 (R)	>16 (R)	16 (R)	>16 (R)	>16 (R)	0.5 (S)	1 (S)	>16 (R)	0.03

*AMX, amoxicillin; CIP, ciprofloxacin; CLI, clindamycin; CRO, ceftriaxone; ERY, erythromycin; I, intermediate; LEV, levofloxacin; LZD, linezolid; MER, meropenem; OFL, ofloxacin; PEN, penicillin; R, resistant; S, susceptible; TET, tetracycline; TIG, tigecycline; VAN, vancomycin. †No susceptibility breakpoints are established for ciprofloxacin and tigecycline.

Most of the 16 isolates in our study were of serotype 11A (n = 9): 5 isolates of ST-9875, 2 of ST-8279, and 1 each of ST-10300 and ST-99. An XDR ST-8279 (serotype 13) clone described in 2014 (*2*) was closely related to the 9 serotype 11A isolates in our study. ST-8279 is a double-locus (*aroE* and *xpt*) variant of ST-156, which is closely related to global clone Spain9V-3 (*2*). Spain9V-3 is related to 3 ST-3642 isolates (serotype 11A) reported in Taiwan in 2010 (*11*) and to 3 MDR ST-166 isolates (serotype 11A) reported in South Korea in 2013 (*12*). In our study, 3 novel STs of MDR *S. pneumoniae* were identified (ST-9875, ST-9876, and ST-10300). All the ST-8279, ST-9875, and ST-10300 isolates in our study were serotype 11A, with the exception of 1 of the ST-8279 isolates. The ST-9875 and ST-10300 isolates were single-locus variants (in the *spi* and *gki* genes, respectively) of ST-8279. ST-9876 is a 1-locus (*aroE*) variant of an ST-3384 (serotype 9V) clone registered in the PubMLST database.

Serotypes 19F and 23F are included in the 13-valent pneumococcal conjugated vaccine (PCV13), but serotype 11A is not included in PCV13. Serotype 11A is, however, included in the 23-valent pneumococcal polysaccharide vaccine (PPSV23). The US CDC currently recommends the PPSV23 for all adults >65 years of age and all persons 2–64 years of age who are at high risk for pneumococcal disease (*13*). Through national vaccine programs in South Korea, since 2013, PPSV23 has been provided to all adults >65 years of age, and since 2014, 10-valent pneumococcal conjugated vaccine or PCV13 have been provided to young children free of charge (*14*).

## Conclusions

In South Korea, serotype 11A was the most predominant serotype of the 16 levofloxacin-resistant and XDR *S. pneumoniae* isolates we found. Seven levofloxacin-resistant *S. pneumoniae* strains were isolated in 2014 alone; the dominant serotype was again 11A (n = 5). All except 1 of these 7 serotype 11A isolates were resistant to the 9 different antimicrobial agents tested. We identified 3 novel STs of MDR serotype 11A *S. pneumoniae* in our study. *S. pneumoniae* serotype 11A isolates with novel STs require careful monitoring to combat the increasing prevalence and diversification of MDR pneumococcal strains, especially those with resistance to fluoroquinolones, β-lactams, and third-generation cephalosporins.
